# Photo-crosslinked HAMA hydrogel with cordycepin encapsulated chitosan microspheres for osteoarthritis treatment

**DOI:** 10.18632/oncotarget.13748

**Published:** 2016-12-01

**Authors:** Chen Xia, Pengfei Chen, Sheng Mei, Lei Ning, Chenyang Lei, Jiying Wang, Jianfeng Zhang, Jianjun Ma, Shunwu Fan

**Affiliations:** ^1^ Department of Orthopaedics, Sir Run Run Shaw Hospital, School of Medicine, Zhejiang University, Hangzhou, China; ^2^ Key Laboratory of Biotherapy of Zhejiang Province, Hangzhou, China

**Keywords:** hydrogel, cordycepin, autophagy, osteoarthritis

## Abstract

Autophagy is a protective mechanism in normal cartilage. The present study aimed to investigate the synergistic therapeutic effect of promotion of chondrocyte autophagy via exposure to cordycepin encapsulated by chitosan microspheres (CM-cordycepin) and photo-crosslinked hyaluronic acid methacrylate (HAMA) hydrogel, with the goal of evaluating CM-cordycepin as a treatment for patients with osteoarthritis. First, we developed and evaluated the characteristics of HAMA hydrogels and chitosan microspheres. Next, we measured the effect of cordycepin on cartilage matrix degradation induced by IL1-β in chondrocytes and an *ex vivo* model. Cordycepin protects cartilage from degradation partly by activation of autophagy. Moreover, we surgically induced osteoarthritis in mice, which were injected intra-articularly with CM-cordycepin and HAMA. The combination of CM-cordycepin and HAMA hydrogel retarded the progression of surgically induced OA. Cordycepin ameliorated cartilage matrix degradation at least partially by inducing autophagy *in vivo*. Our results demonstrate that the combination of cordycepin encapsulated by CMs and photo-crosslinked HAMA hydrogel could be a promising strategy for treating patients with osteoarthritis.

## INTRODUCTION

Osteoarthritis (OA) is becoming more problematic as the population ages. Patients with OA experience variable degrees of inflammation and degeneration of the articular cartilage, ultimately resulting in exposure of the underlying bone, pain, and disability [1, 2]. Structure-modifying medications and nutraceuticals may be effective therapeutic agents for OA and merit further investigation [3].

Autophagy is the physiological cellular process through which intracellular components undergo lysosome-mediated self-digestion and recycling, and this process is essential for survival, differentiation, development, and homeostasis [4, 5]. Meanwhile, autophagy is a protective mechanism in normal cartilage [6]. Reduced expression of autophagy regulators was observed in pathological cartilage in humans and mice [7]. Activation of autophagy by rapamycin treatment reduced the severity of OA in experimental models [8]. Therefore, autophagy activation may be a novel therapeutic target for OA treatments. Microtubule-associated protein 1A/1B-light chain 3 (LC3) is a soluble protein that is distributed ubiquitously in mammalian tissues and cultured cells [9]. During autophagy, LC3 is recruited to the autophagosomal membrane [10].

Cordycepin(3′-deoxyadenosine), a derivative of the nucleoside adenosine, is one of the major bioactive components of Cordycepsmilitaris [11]. Various studies have focused on the pharmacological activities of cordycepin and revealed it exerted several properties, such as anti-inammatory [12, 13], anti-angiogenesis [14], anti-aging [15], anti-tumor [16]. These evidences suggested that cordycepin have important roles in clinical application. In the development of OA, adisintegrin and metalloproteinase with thrombospondin motifs-5 (ADAMTS-5) and matrix metalloproteinase 13 (MMP13) have been identified as the major enzymes responsible for the cartilage degradation [17, 18]. Moreover, a recently study has found that cordycepin inhibited expressions of ADAMTS-5 and MMP13 in IL-1β-induced osteoarthritis (OA), indicating cordycepin may be a potential candidate to prevent inflammation of OA [19]. In addition, previous researches have found that cordycepin can induces apoptosis and autophagy [20, 21]. These clues suggest that cordycepin protects chondrocytes by facilitating autophagy and preventing cartilage degradation. However, few investigations have been carried out to evaluate the therapeutic efficacy of cordycepin as a treatment for patients with OA.

Chitosan microspheres (CMs) have been established as a useful tool for drug delivery, as they are easy to prepare and can release drugs in a controlled and/or sustained manner, including in OA [22]. CMs are characterized by biocompatibility, nontoxicity, non-allergenic, and biodegradability; as a result, CMs can successfully provide site-specific drug delivery [23]. In this study, the hyaluronic acid methacrylate (HAMA) hydrogel was used as a vehicle to facilitate intra-articular injection of chitosan. Compared with other hydrogels, HAMA can be synthesized at different methacrylation degrees to fabricate hydrogels with tunable physical properties including degradation, stiffness, and pore architecture, and show promise for tissue engineering applications [24]. However, thus far, no investigation has been carried out to evaluate its therapeutic efficacy in animal models of OA, making its suitability for this purpose uncertain. In this study, we report the results of experiments in which HAMA hydrogels containing chitosan microspheres encapsulating cordycepin were injected into mouse joints, with the goal of producing sustained cordycepin release and ameliorated OA changes.

To assess the therapeutic potential of microsphere-encapsulated cordycepin as a treatment for patients with OA, we developed and evaluated the characteristics of a photo-crosslinked HAMA hydrogel, after which chitosan microspheres were utilized to produce controlled cordycepin release. Moerover, we investigated the effects of cordycepin on OA cartilage degeneration. We hypothesized that the combination of chitosan microsphere-encapsulated cordycepin (CM-cordycepin) with our newly developed photo-crosslinked HAMA hydrogel would synergistically prevent the progression of degenerative changes in surgically induced OA.

## RESULTS

### Preparation and characterization of HAMA hydrogels and chitosan microspheres

**Figure 1:**
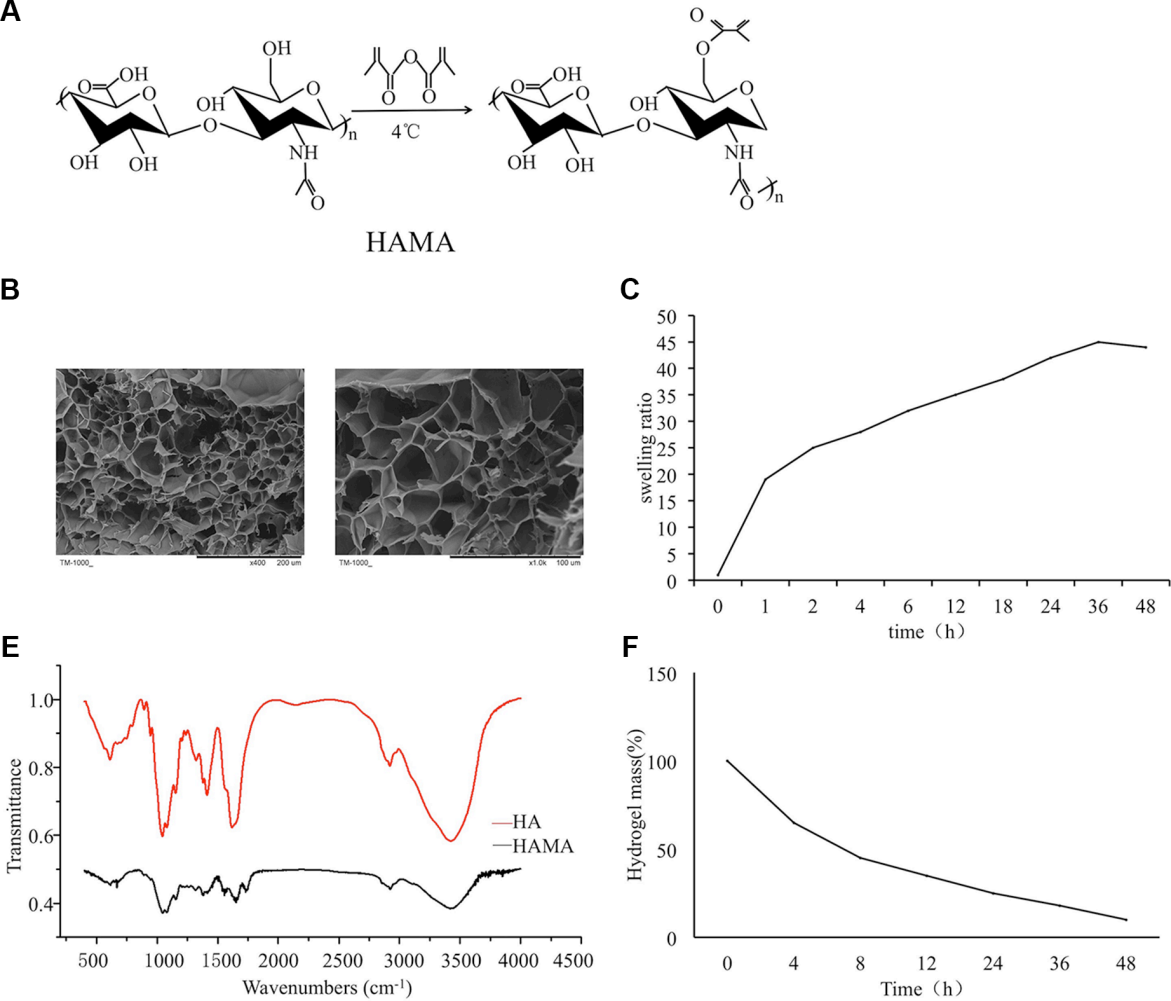
Preparation and characterization of HAMA hydrogels (**A**) Hyaluronic acid containing primary hydroxy groups were reacted with methacrylic anhydride (MA) to add methacrylate pendant groups. (**B**) Microscopic structure of the hydrogel. (**C**) Swelling kinetics of the hydrogel. (**D**) *In vitro* degradation profile of the hydrogel. (**E**) Fourier transform infrared (FTIR) spectroscopy for HAMA.

The HAMA hydrogels were formed by exposing the prepolymer to UV light for 2 minutes. Scanning electron microscopy of the gel cross-sections showed the porosity of the gels. Disordered pores of 50–150 μm were uniformly distributed throughout the gel. Hyaluronic acid containing primary hydroxy groups was reacted with methacrylic anhydride (MA) to add methacrylate pendant groups (Figure 1A). The HAMA hydrogels showed a highly inter-connective porous structure, honeycomb-like structure (Figure 1B). The swelling ratios of the hydrogels in PBS are shown in Figure 1C. The hydrogels exhibited fast swelling and reached equilibrium within 36 h. In tests of the degradation profile of the crosslinked hydrogel *in vitro*, approximately 10.8% of the hydrogel remained after 48 hours (Figure 1D). The FTIR spectra of HAMA and HA are shown in (Figure 1E). HAMA showed a new peak at 1731 cm^−1^, which can be ascribed to the added C=O groups. The appearance of new C=C groups attributed to a new peak at 1643 cm^-1^. FTIR spectroscopy result demonstrated that hyaluronic acid was reacted with methacrylic anhydride (MA) to add methacrylate pendant groups (Figure 1E).

**Figure 2:**
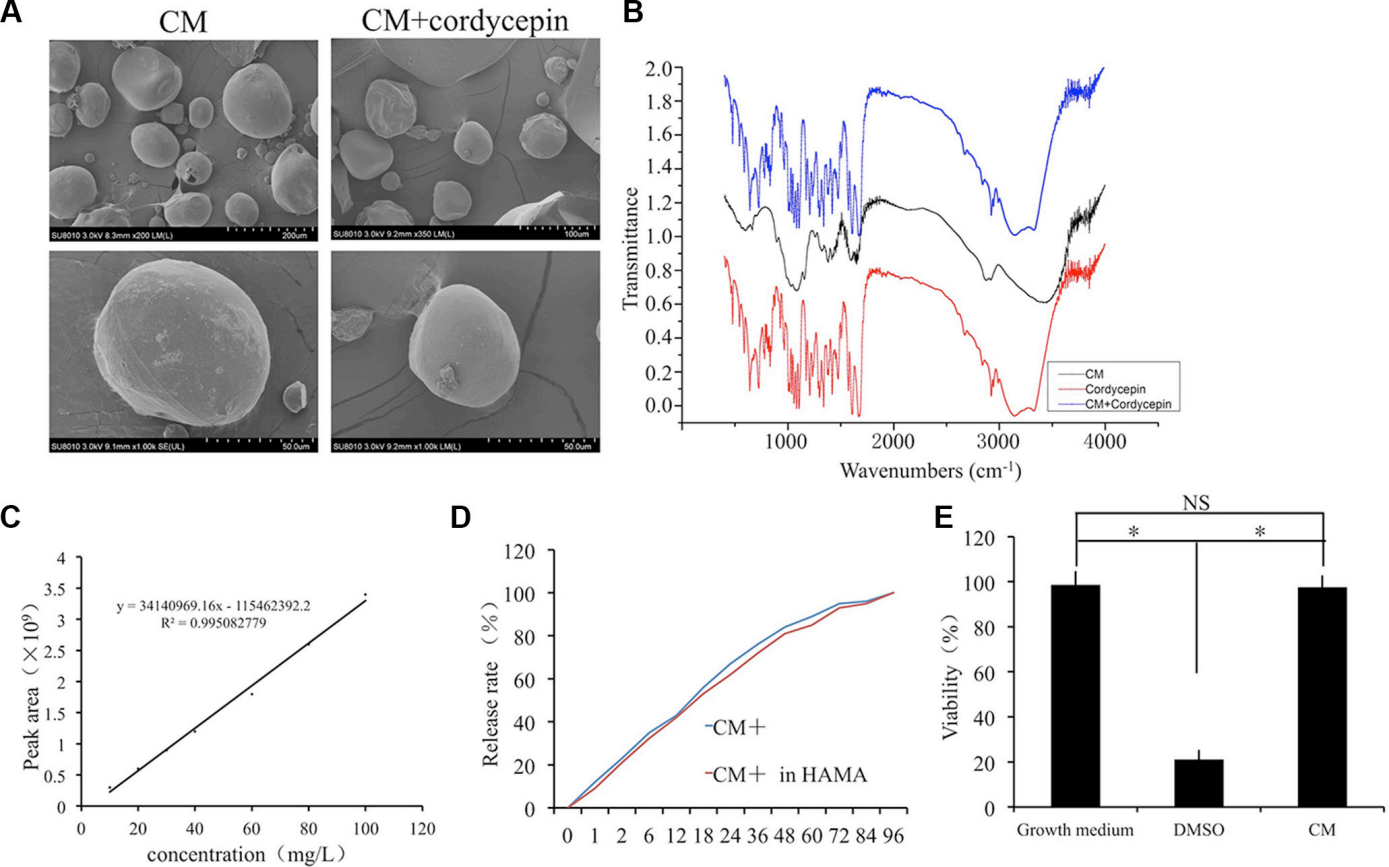
Preparation and characterization of chitosan microspheres (**A**) Scanning electron micrographs of unloaded chitosan microspheres (CM) and cordycepin-loaded chitosan microspheres (CM-cordycepin). (**B**) Fourier transform infrared (FTIR) spectroscopy for chitosan microspheres with cordycepin. (**C**) Standard curve of cordycepin. (**D**) Controlled release profile of chitosan microspheres. (**E**) Cytotoxicity test of the chitosan microspheres.

Next, CMs and cordycepin-loaded CMs were characterized by SEM. Drug loading did not significantly change the structure of the microspheres. The CMs had an orbicular shape, a mean size of approximately 100 μm, and a smooth outward appearance without cracks (Figure 2A). Cordycepin shows characteristic peaks at 1673 cm^−1^, 1609 cm^−1^, which can be attributed to C=N and -NH_2_. The Characteristic peak of the 3140 cm^-1^ was observed due to -OH. And CM-cordycepin shows same characteristic peaks in the FTIR spectra, which demonstrated that cordycepin was encapsulated into chitosan microspheres (Figure 2B). We tested the chitosan microspheres with cytotoxicity tests. The cell viability results showed that the chitosan microspheres were not toxic (Figure 2E). The EE was 90.58 ± 6.43%, and the chitosan microspheres showed 1.89 ± 0.21% DL, indicating that most of the drug was encapsulated in the microspheres. The controlled release property of the CMs was determined by HPLC (Figure 2C). In the cordycepin-loaded CMs without hydrogel group, approximately 50% of the CM-encapsulated cordycepin was released at a relatively stable rate during the first 24 h, after which the release rate slowed. All of the CM-encapsulated cordycepin was released within 72 h in PBS (Figure 2D). There was no significant difference in the release rate of cordycepin between cordycepin-loaded CMs with and without hydrogel (Figure 2D).

### Cordycepin ameliorates cartilage degeneration through autophagy

**Figure 3:**
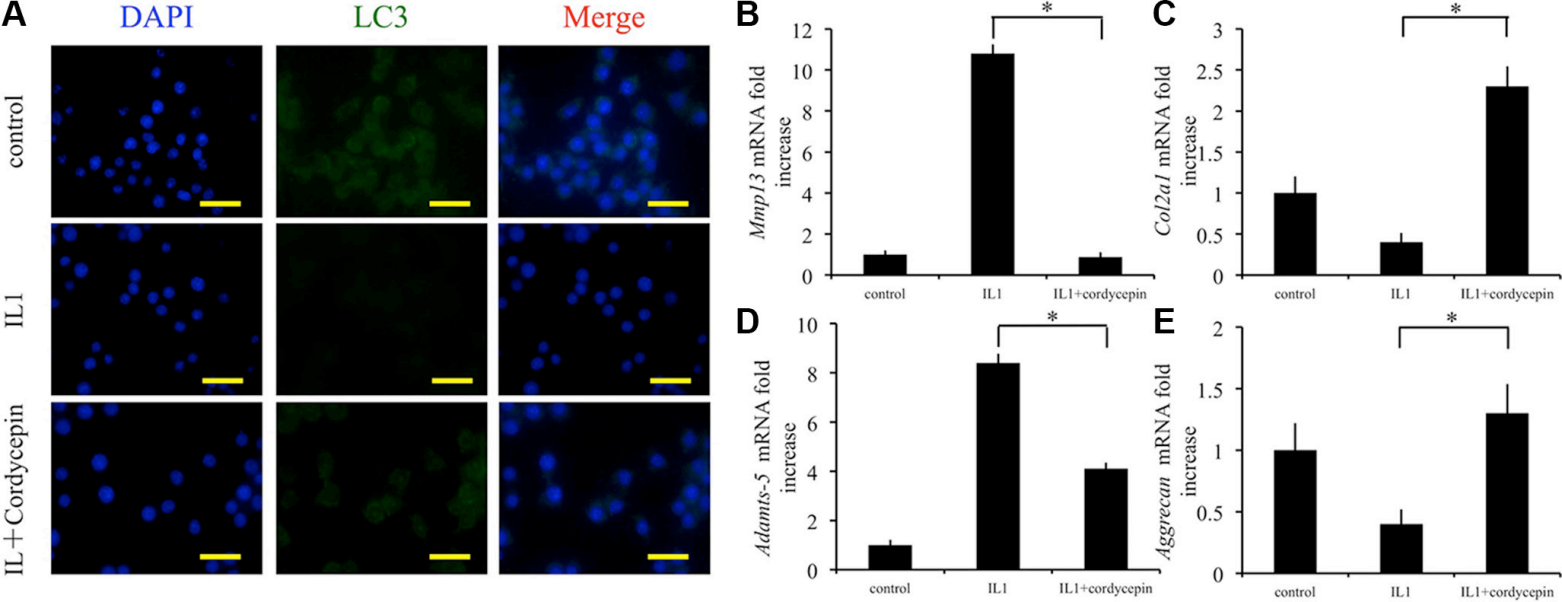
Cordycepin ameliorates cartilage degeneration by regulating autophagy (**A**) Immunocytochemistry to detect LC3 in chondrocytes. Scale bars = 30 μm. (**B**–**E**) The effects of cordycepin on mRNA transcript levels of *Mmp13, Col2a1, Adamts-5* and *Aggrecan* after treatment with IL-1 (10 ng/mL) for 48 h *n* = 3, *p < 0.05 (one way ANOVA).

We verified the protective effect of cordycepin against OA cartilage degeneration and investigated the mechanisms underlying this activity. In 10 ng/mL IL1-β-stimulated chondrocytes, cordycepin decreased mRNA expression of *Mmp13* and *Adamts-5*, whereas it increased mRNA expression of *Col2a1* and *Aggrecan* (Figure 3B–3E). In addition, we evaluated LC3 expression by measuring immunofluorescence. In comparison with IL1-β treated chondrocytes, chondrocytes treated with IL1-β and cordycepin exhibited a higher level of LC3 expression (Figure 3A). These results indicate that activation of autophagy by cordycepin ameliorates IL1-β induced pathological changes characteristic of OA.

### Effect of cordycepin on cartilage matrix degradation *ex vivo*

**Figure 4:**
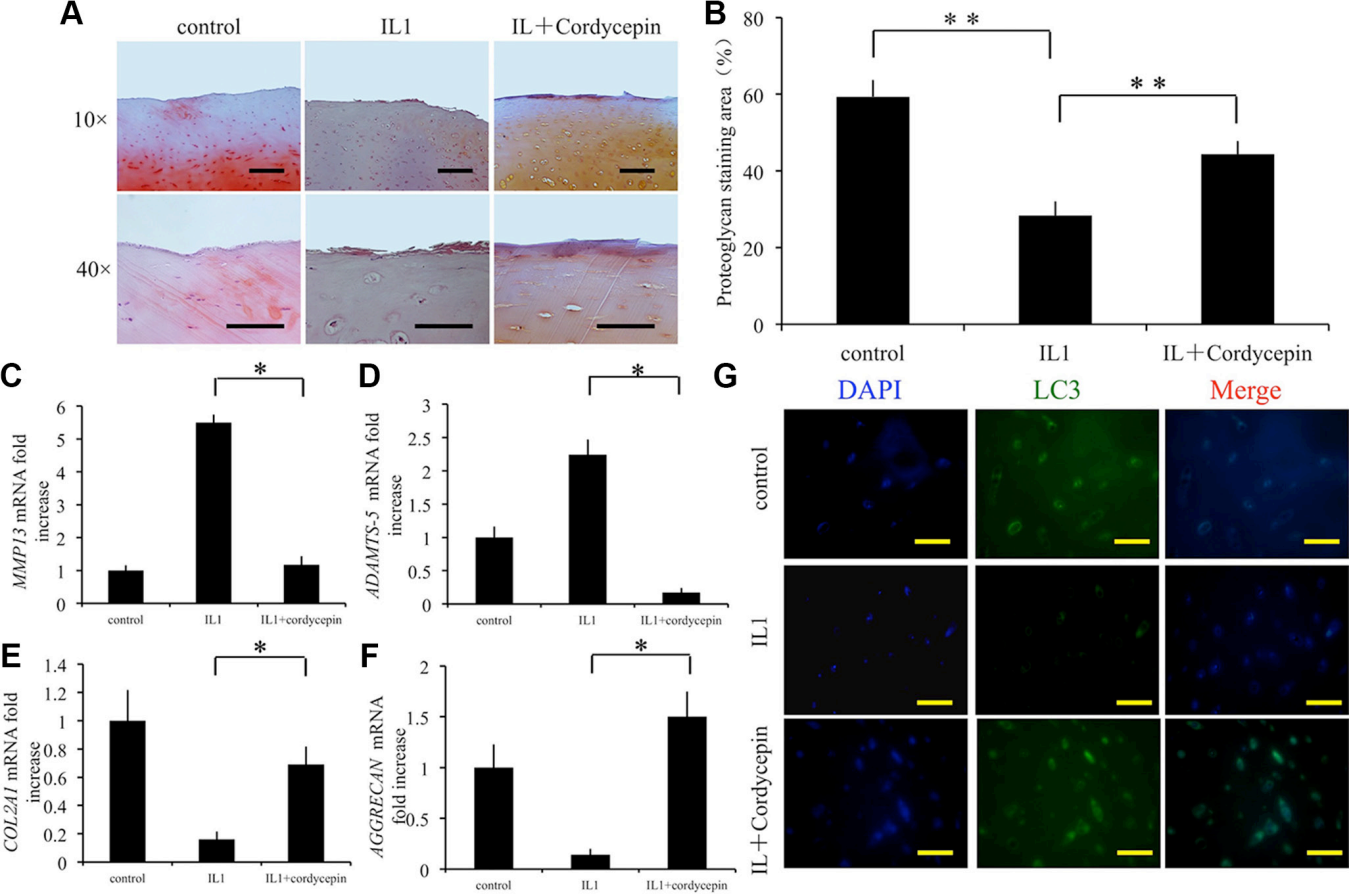
Effect of cordycepin on cartilage matrix degradation in an ex vivo model (**A**, **B**) Cordycepin significantly attenuated loss of proteoglycan in cartilage induced by IL-1, assayed by Safranin O staining. Cartilage samples were isolated from patients with OA and cultured with 10 ng/mL IL-1, in presence or absence of 100 μM cordycepin(**C**–**F**). The effects of cordycepin on mRNA transcriptlevels of *MMP13, COL2A1, ADAMTS-5, and AGGRECAN*. *n* = 3, *p < 0.05 (one way ANOVA). (**G**) Immunocytochemistry was performed to assess expression of LC3. Scale bars = 100 μm. *n* = 3, *p < 0.05 (one way ANOVA).

The effect of cordycepin was evaluated *ex vivo* in OA cartilage explants from human. Knee-joint cartilage was placed in explant culture medium and stimulated with IL1-β to induce matrix degradation. Separate groups of samples were treated with IL1-β, or IL1-β with cordycepin for 2 days. The extent of matrix degradation was assessed by safranin-O staining. In comparison with IL1-β-treated cartilage, cartilage that was simultaneously treated with IL1-β and cordycepin exhibited more safranin O-positive proteoglycan (Figure 4A, 4B). Quantitative analysis demonstrated that cordycepin significantly decreased mRNA expression of *MMP13* and *ADAMTS-5* compared to the tissue exposed to IL1-β only (*p* < 0.05) (Figure 4C, 4D). In addition, cordycepin significantly increased expression of cartilage extracellular matrix components *COL2A1* and AGGRECAN (*p* < 0.05) (Figure 4E, 4F).

To determine the mechanism involved in the effect of cordycepin on cartilage degradation, we measured expression of autophagy marker LC3 by immunofluorescence. Simultaneous treatment with IL1-β and cordycepin significantly increased LC3 positive chondrocytes in cartilage tissue in comparison with that of the tissue exposed to IL1-β only (*p* < 0.05) (Figure 4G). These results indicate that cordycepin protects cartilage from degradation partly by activation of autophagy.

### Efficacy of the combination of CM-cordycepin with HAMA hydrogel as an OA treatment

**Figure 5:**
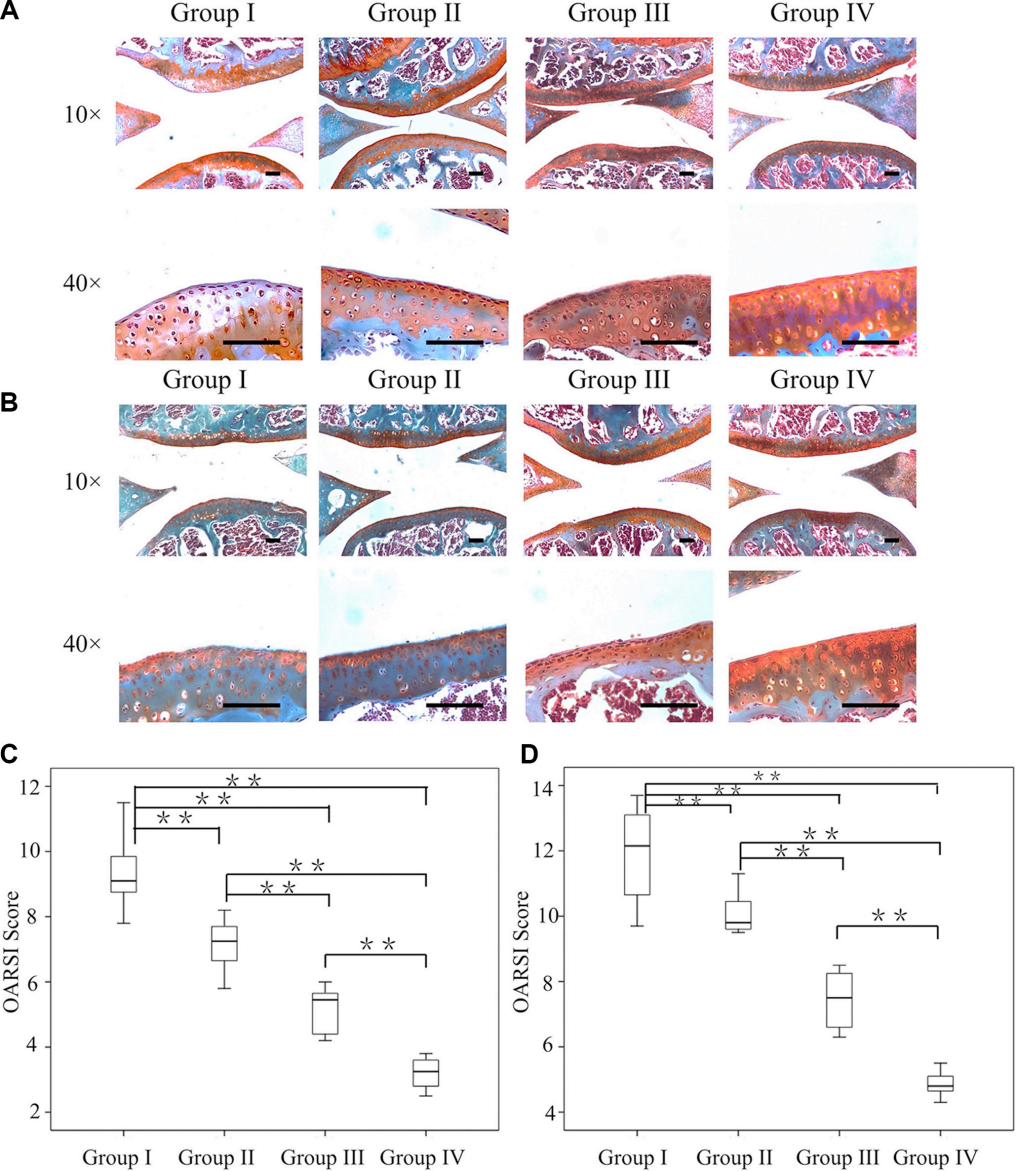
Efficacy of the combination of CM-cordycepin and HAMA hydrogel as a treatment for OA Safranin-O staining of cartilage samples 4 weeks (**A**) and 8 weeks (**B**) after OA induction. Scale bars = 100 μm. (**C**, **D**) OARSI scores of samples 4 and 8 weeks after OA induction. *n* = 8, **p < 0.01 (Kruskal-wallis ANOVA). Group I, OA mice; group II, OA mice treated with HAMA hydrogel; group III, OA mice treated with cordycepin; group IV, OA mice treated with CM-cordycepin and HAMA hydrogel.

CM-cordycepin with hydrogel was intra-articularly administered to mice to determine whether it delayed the progression of OA. 4 and 8 weeks after surgery, immunohistochemical staining and OARSI scoring were used to evaluate the joints of the mice. Four weeks after surgery, In comparison with OA mice (group I), OA mice treated with HAMA alone (group II) showed significant differences in terms of the OARSI score. Meanwhile, the OA mice treated with cordycepin alone (group III) showed improved cartilage surfaces and significantly lower OARSI scores compared to the mice in group I after 4 weeks (*p* < 0.01). Interestingly, the progression of OA was retarded to the greatest extent inthe mice that received CM-cordycepin + HAMA hydrogel (group IV), as evidenced by reduced cartilage degradation, more safranin-O staining (Figure 5A), and the lowest OARSI score (3.4 ± 0.3) among the groups with surgically induced OA (Figure 5C).

Eight weeks after surgery, group I showed an ossified joint surface, decreased safranin-O staining, and the highest OARSI score among the groups (12.7 ± 1.0, Figure 5B and 5D). In comparison with group I, group II and group III showed significantly better surface histology and a lower OARSI score (*p* < 0.01). Notably, the mice in group IV had the best joint surfaces among the experimental groups and the lowest OARSI score (4.5 ± 0.3) (Figure 5B, 5D).

These results indicate that treatment with the combination of CM-cordycepin and HAMA hydrogel retarded the progression of surgically induced OA, while each of these components alone also had a mild beneficial effect.

### Effect of cordycepin on cartilage matrix degradation *in vivo*

**Figure 6:**
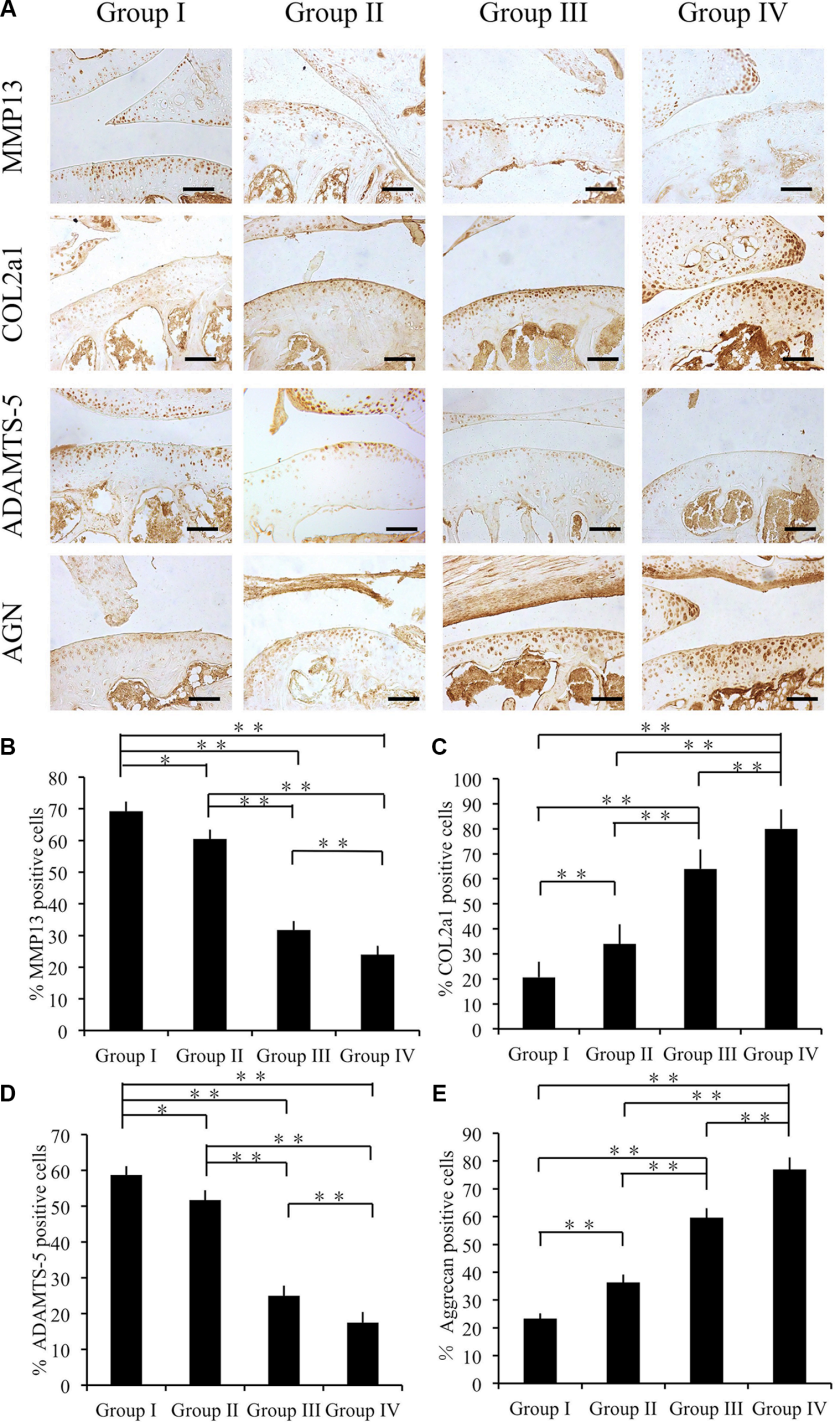
Effect of cordycepin on cartilage matrix degradation in vivo (4 weeks) (**A**) Immunohistochemistry of MMP13, COL2A1, ADAMTS-5, and AGGRECAN. Scale bars = 100 μm. (**B**–**E**) Quantification of MMP13-positive (**B**), COL2A1-positive (**C**), ADAMTS-5-positive (**D**) and AGGRECAN-positive-cells (**E**) within cartilage samples at 4 weeks postsurgery. *n* = 8, **p < 0.01 (one way ANOVA).

In the pathological process of OA, events such as cartilage calcification and degradation often occur; therefore, we determine the capacity of cordycepin to ameliorate these events using an immunohistochemical assay to measure cartilage degradation marker (MMP13, COL2A1, ADAMTS-5, and AGGRECAN) expression. In comparison with group I, group II, group III and group IV showed significantly fewer chondrocytes expressing MMP13 and ADAMTS-5 after 4 weeks (*p* < 0.01). Also group IV showed reductions in MMP13 and ADAMTS-5 expression levels in comparison with those of group III (*p* < 0.05) (Figure 6A, 6B, 6D). In addition, group I had significantly lower COL2A1- and AGGRECAN-positive chondrocytes among all the groups (*p* < 0.05) (Figure 6A, 6C, 6E).

**Figure 7:**
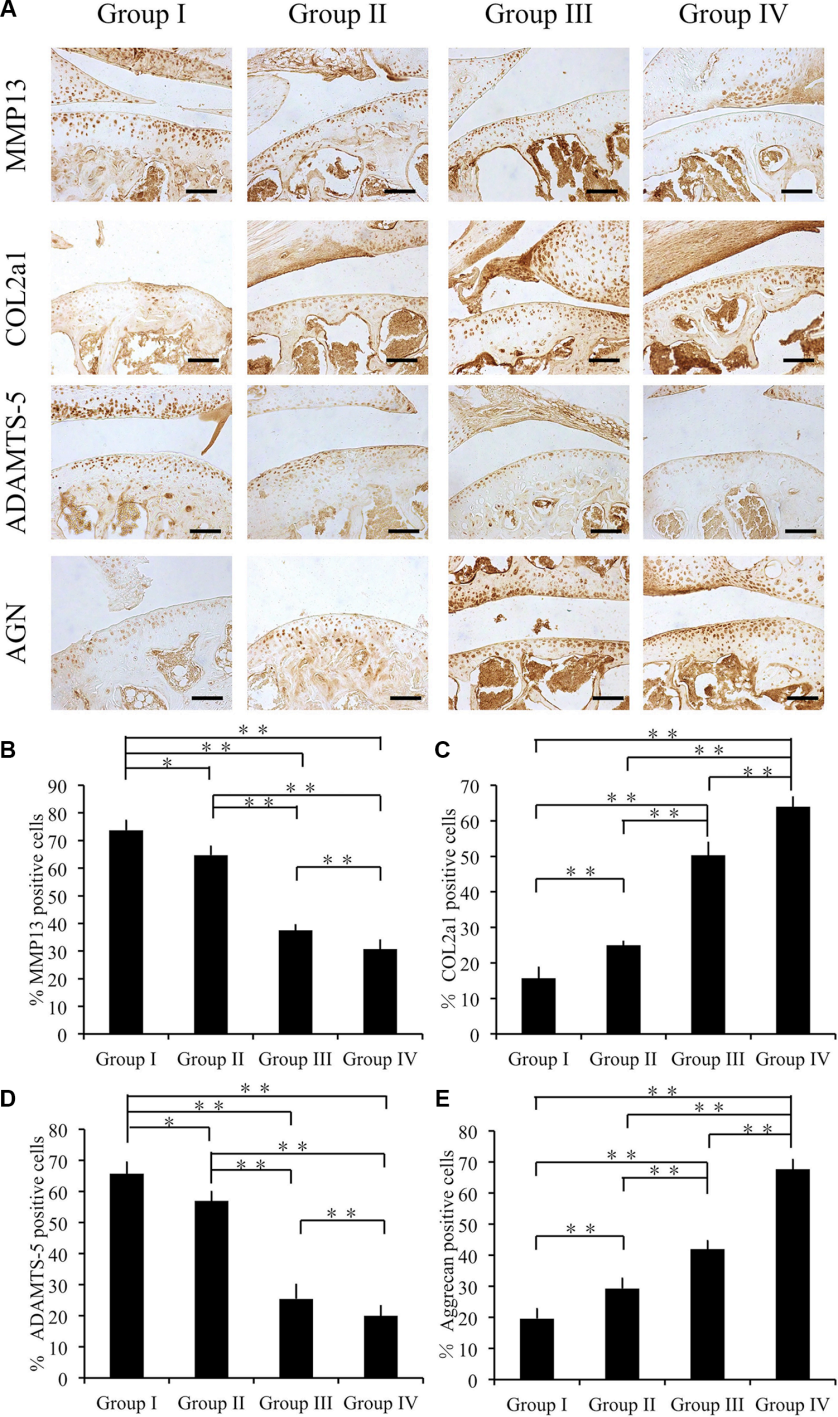
Effect of cordycepin on cartilage matrix degradation in vivo (8 weeks) (**A**) Immunohistochemistry of MMP13, COL2A1, ADAMTS-5, and AGGRECAN. Scale bars = 100 μm. (**B**–**E**) Quantification of MMP13-positive (**B**), COL2A1-positive (**C**), ADAMTS-5-positive (**D**) and AGGRECAN-positive-cells (**E**) within cartilage samples at 8 weeks postsurgery. *n* = 8, **p < 0.01 (one way ANOVA).

Eight weeks after surgery, group I showed the highest expression levels of MMP13 and ADAMTS-5 compared with group II, group III and group IV (*p* < 0.01). In comparison with group I, group II and group III showed significant differences in expression levels of MMP13. Notably, group IV showed the lowest expression levels of MMP13 and ADAMTS-5 among the experimental groups (*p* < 0.05) (Figure 7A, 7B, 7D). Remarkably, the COL2A1 and AGGRECAN expression levels of group II, group III and group IV were significantly higher than those of group I (*p* < 0.05) (Figure 7C, 7E).

These results indicate that treatment with the combination of CM-cordycepin and HAMA impedes cartilage degradation.

### Effect of cordycepin on chondrocyte autophagy *in vivo*

**Figure 8:**
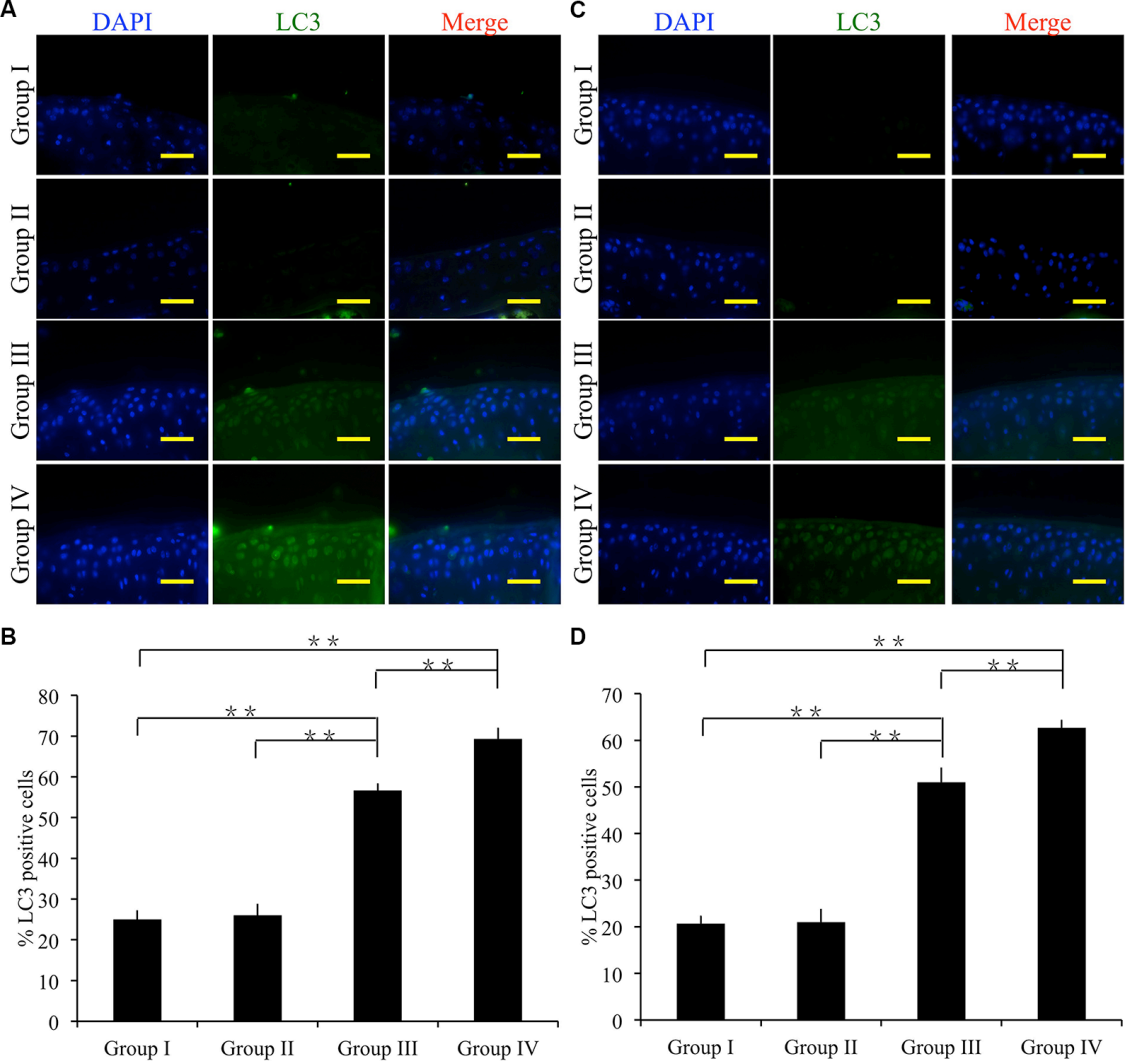
Effect of cordycepin on chondrocyte autophagy in vivo (**A**, **B**) Immunocytochemistry was performed for LC3 after 4 weeks. (**C**, **D**) Immunocytochemistry was performed for LC3 after 8 weeks. Scale bars = 100 μm. *n* = 8, **p < 0.01 (one way ANOVA).

To determine the mechanism involved in the therapeutic effect of cordycepin, we measured expression levels of autophagy marker LC3 by immunofluorescence. Four weeks after surgery, group III, group IV showed significantly more chondrocytes expressing LC3 than did group I (*p* < 0.01). However, group II showed no significant difference compared with group I (Figure 8A, 8B). Group IV showed significantly more LC3 positive chondrocytes than did group III (Figure 8A, 8B) (*p* < 0.01).

Eight weeks after surgery, group I showed the fewest LC3 positive chondrocytes compared with group III, group IV (*p* < 0.01). In comparison with group I, group II showed no significant difference in the number of LC3 positive chondrocytes. Notably, group V showed the greatest number of LC3 positive chondrocytes among the experimental groups (*p* < 0.01) (Figure 8C, 8D).

These results demonstrate that cordycepin inhibits cartilage matrix degradation *in vivo* partly by enhancing autophagy.

## DISCUSSION

In this study, we developed a new strategy for treating OA, in which CM-cordycepin combined with HAMA hydrogel were applied. The three major findings of this study were as follows: first, we found that cordycepin ameliorated cartilage degeneration in *ex-vivo* models; second, cordycepin enhanced autophagy in mouse chondrocytes and human OA cartilage; third, the combination of CM-cordycepin and HAMA hydrogel slowed the progression of degenerative changes in OA.

During the development of OA, autophagy may increase as an adaptive response to protect cells from various stresses, and failure of the adaptation may lead to further progression of degeneration [25]. Previous research on human OA joints and joints from experimental OA models demonstrated reduced expression of autophagy regulators in association with increased cell death [7]. It was confirmed that the induction of autophagy caused OA-like gene (Aggrecan, Col2a1, Mmp13, and Adamts5) expression changes through the modulation of apoptosis and reactive oxygen species [25]. MMP13 and ADAMTS-5 are regarded as the major enzymes responsible for degradation of the cartilage matrix. We found that cordycepin can effectively induct autophagy and ameliorated cartilage degeneration by decreasing MMP13 and ADAMTS-5 expression. Thus, we concluded that cordycepin regulated degradation of cartilage matrix via the induction of autophagy.

HA has many unique properties, including its biocompatibility, viscoelasticity, and lack of immunogenicity. And HA is one of the major ingredients of cartilage ECM and is involved in cell proliferation, morphogenesis, inflammation, and wound repair [26]. Moreover, HA-based materials are emerging as a cell scaffold platform for tissue engineering applications, including the creation of tissue-engineered bone or cartilage [27]. In this study, HA was modified to form photopolymerizable hydrogels, and this hydrogels showed *in vitro* degradation properties that supported their suitability for cartilage tissue engineering. As an intra-articular-injected material, it also possessed a high swelling ratio needed to cover the articular surface. These properties make this hydrogel a suitable material for cartilage tissue engineering. In our *in vivo* experiment, the HAMA hydrogel retarded the progression of surgically induced OA compared to that in untreated OA mice at 4 and 8 weeks. This may because of the ECM ingredients properties of HAMA. Thus, we suggest that HAMA has a protective effect on articular cartilage.

CMs are regarded as an attractive biopolymer for applications requiring sustained and controlled release of drug [28]. Although cordycepin delivered via these routes plays a therapeutic role [29], intra-articular injection is regarded as the most common and effective method of drug delivery in OA [30, 31]. In this study, the effect of intra-articular injected cordycepin was further improved due to the controlled release property of CMs. The combination of CM-cordycepin and HAMA hydrogel was injected into mouse joints to produce sustained cordycepin release and ameliorate degenerative changes in osteoarthritic cartilage. CM-cordycepin was intra-articularly injected once a week, however because of the relative inhomogeneity and large size of CMs, the controlled release system used in this study was only capable of sustained release for 3 days. Future studies will be aimed at developing more efficient drug delivery systems by controlling CM size and homogeneity, with the goal of maintaining therapeutic drug concentrations in the joints of patients with OA. In our experiment, group III received intra-articular injections with cordycepin alone (5 mg/kg). We found that intra-articular injection with cordycepin alone also suppressed the progression of surgically-induced OA. Periarticular drug injections and intraperitoneal injections have difficulty reaching the lesion site. However, intra-articular injections may also increase OA damage. Thus, other local injection methods should be investigated.

## MATERIALS AND METHODS

### Materials

Methacrylic anhydride (MA) was purchased from Sigma-Aldrich (St. Louis, MO, USA), HA (MW = 350 kDa) was purchased from Lifecore Biomedical (Chaska, MN, USA). All other chemicals were purchased from Sigma-Aldrich (St. Louis, MO, USA) unless specifically mentioned.

### Methacrylated hyaluronic acid synthesis

HAMA was also synthesized following a previously described procedure [36]. Briefly, 1 g hyaluronic acid sodium salt was dissolved in 100 mL distilled water until it fully dissolved. MA was then added to this solution at 1% (v/v) and the reaction was performed for 24 h at 4°C by maintaining the pH between 8–10 with the addition of 5 M sodium hydroxide. The resulting solution was dialyzed in a 12–14 kDa dialysis membrane at 4°C for 3 days, frozen at −80°C, and freeze dried to obtain a solid product, which was then kept at −80°C until further use.

### Hydrogel preparation

The prepolymer hydrogel solution was prepared by mixing 2 wt% HAMA solution into PBS containing 1% (w/v) 2-hydroxy-1- (4-(hydroxyethoxy)phenyl)-2-methyl-1-propanone (Irgacure 2959, CIBA Chemicals) as a photoinitiator at 80°C. The HAMA prepolymer solution was vigorously stirred at room temperature for 10 minutes to generate a homogeneous solution, which was pipetted into a 24-well culture dish (200 μL/well) and exposed to UV light (320–500 nm, 7.0 mW/cm^2^) for 2 minutes to allow for photo-crosslinking. The samples were incubated in a free-floating manner at 37°C in PBS for 24 h, followed by storage at −20°C.

### Characterization of HAMA hydrogel

The hydrogel swell ratio was analyzed as follows. The hydrogels were lyophilized until dry. To ensure the success of the reaction, HAMA and MA were characterized by Fourier transform infrared (FTIR) spectroscopy (Nicolet AVA TAR370, Thermo Scientific, Waltham, MA, USA). Dry weight (Wd) was measured. Dried hydrogel samples (*n* = 3) were immersed in 50 mL of PBS at 37°C and allowed to swell. Swollen hydrogel samples were weighed to determine swollen weight (Ws) at different time points. The swelling ratio (Q) was calculated by the following equation: *Q* = Ws/Wd.

To characterize the enzymatic degradation properties, we placed the HAMA hydrogel samples in 1.5-mL centrifuge tubes with 1 mL PBS containing 10 units of hyaluronidase at 37°C. At a pre-defined time (Supplementary Table S1), the hydrogels for each condition were removed, frozen, and lyophilized. Mass loss was determined as the ratio of the final weight to the original dry weight. All experiments were repeated 3 times.

### Preparation of chitosan microspheres

Chitosan microspheres were prepared using the water-in-oil (W/O) emulsion solvent diffusion method. Chitosan solution (2% w/v) was prepared by dissolving chitosan (Shanghai Bio Science and Technology) in 2.5% (v/v) acetic acid aqueous solution (Sinopharm Chemical Reagent) at room temperature. The chitosan solution was mixed with cordycepin by stirring overnight with a magnetic stirrer to produce a homogeneous mixture. Next, 5 mL of the resulting mixture was aspirated into a syringe pump and added drop-wise into the oil phase (24.72 mL), which consisted of 14 mL liquid paraffin (Sinopharm Chemical Reagent), 10 mL petroleum ether, and 0.72 mL Span 80 (Sangon Biotech), at a flow rate of 4 mL/h with continuous stirring at 1500 rpm. A syringe needle with an internal diameter of 0.2 mm was used for this process. After the solvent diffusion procedure, the suspension was cross-linked using 25% (v/v) glutaraldehyde solution as a cross-linking agent. The addition of the cross-linker was carried out three times, at time intervals of 15 min, with the following volumes of glutaraldehyde: 0.64, 0.64, and 0.32 mL. Subsequently, the suspension was stirred at room temperature to produce cross-linking and centrifuged at 3000 rpm for 5 min, after which the supernatant fluid was discarded. Next, the microspheres were washed with petroleum ether (three times) (Sinopharm Chemical Reagent), methanol (two times) (Sinopharm Chemical Reagent), acetone (one time) (Sinopharm Chemical Reagent), isopropyl alcohol (one time) (Sinopharm Chemical Reagent), ethanol (one time), and distilled water (three times). After washing, the microspheres were collected after lyophilization with a freeze-dryer to remove residual water. For the control group, pure chitosan microspheres were prepared by directly dropping the chitosan solution into the oil phase under the same conditions. We embedded the CM in the HAMA hydrogel before photo-crosslinking. To ensure that cordycepin was encapsulated into chitosan microspheres after lyophilizing, the prepared chitosan microspheres containing cordycepin was characterized by Fourier transform infrared (FTIR) spectroscopy (Nicolet AVA TAR370, Thermo Scientific, Waltham, MA, USA).

### Encapsulation efficiency and drug loading

The drug encapsulation efficiency (EE) and drug loading (DL) were determined. Specific amounts of CM-cordycepin and buffer solution were added to centrifuge tubes, and the tubes were centrifuged at 5,000 rpm for 15 min. The amount of free drug in the filtrate was determined by HPLC. Then, 1% hydrochloric acid was added to destroy the chitosan microspheres. The amount of total drug in the liquid was determined using HPLC. The EE and DL were calculated using Eqs. 1 and 2:

where W_total_, W_free_, and W_CM_ represent the total amount of drug in the chitosan microspheres, the amount of drug in the liquid, and the amount of chitosan microspheres, respectively.

### Cell viability assay

The 1 mg chitosan microspheres were immersed in 1 ml DMEM-high glucose containing 10% FBS and 1% penicillin/streptomycin for > 24 h. BMSCs were seeded on a 96-well plate at 10^3^ cells/well. After 24 h, the medium of the test group was replaced with leaching liquor acquired from chitosan microspheres or fresh DMEM-high glucose containing 10% FBS and 1% penicillin/streptomycin or 5% dimethyl sulfoxide (DMSO). After 3 days, the cytotoxicity of the chitosan microspheres was assessed using Cell Counting KIT-8 (CCK-8, Dojindo, Kumamoto, Japan) at 450 nm. Cell number was correlated with optical density (OD).

### *In vitro* cordycepin release profile

To characterize the standard curve of cordycepin, a suitable amount of cordycepin was weighed to prepare different concentrations of cordycepin solutions (5, 10, 20, 30, 40, 60, 80, 100 and 120 mg/L). The quantitative analysis of cordycepin was performed using HPLC (Nexera, Shimadzu Corporation, Kyoto, Japan) equipped with a diode array detector (Shimadzu model SPD-M20A). Cordycepin was eluted through a reverse phase Diamonsil column, 5-μm particle size, 250 mm × 4.6 mm (Agilent, Santa Clara, CA, USA) at a flow rate of 0.8 ml/min. The injection volume was 10 μl. A mixture of acetonitrile and water (80:20) was used as a mobile phase. The amount of cordycepin was determined at a wavelength of 260 nm. At concentrations ranging between 20–100 mg/L, the standard curve showed a good linear relationship (Figure 2). *In vitro* release profiles from cordycepin-loaded CMs, and cordycepin-loaded CMs with hydrogel were determined at pH 7.4 with 0.01 M phosphate-buffered saline solution. The amount of cordycepin, CMs and hydrogel was 0.4 mg, 100 mg, and 40 μL, respectively. The cordycepin-loaded CMs with or without hydrogel were placed in a 50 ml Eppendorf tube. Then, 20 ml 0.01 M PBS was added into the tube. The sample was incubated at 37°C with gentle agitation. At the desired times (0, 1, 3, 6, 12, 18, 24, 36, 48, 60, 72 h), 10 μl solutions were quantified by HPLC. The experiments were carried out in triplicate.

### SEM characterization

To characterize the internal microstructures of the materials, the swollen hydrogel samples and chitosan microspheres were frozen at −80°C and lyophilized. The dried samples were mounted on aluminum stubs, sputter-coated with gold, and observed under a scanning electron microscope (Hitachi S3000N) at an accelerating voltage of 15 kV.

### Human cartilage and primary cultures of mouse chondrocytes

The patients’ consent as well as approval of the local ethics committee was obtained prior to harvesting of human tissue samples. Control cartilage was obtained from 6 OA patients undergoing total knee replacement surgery.

Eight mouse articular cartilage samples were obtained from the femoral condyles and tibial plateaus of C57BL/6 mice on postnatal day 5–6. Cartilage specimens were cut into small slices and minced, and then washed three times with sterile PBS. Then, the chondrocytes were isolated with 0.25% collagenase (Sigma Chemical Co.) in DMEM supplemented with 10% fetal bovine serum (FBS) (Invitrogen Corp., Carlsbad, CA, USA), 1% penicillin/streptomycin, and 0.25 mg/ml fungizone (BioWhittaker, Walkersville, MD, USA) for 18 h at 37°C in a culture plate for digestion. Finally, the isolated cells were collected by centrifugation (1000 rpm, 5 min) and washed three times with the culture medium [35]. The suspended cells were cultured in DMEM supplemented with 10% FBS and 1% penicillin/streptomycin. The cells were maintained at 37°C in a humidified atmosphere with 5% CO_2_ and the culture medium was changed every 2–3 days.

### Quantitative real-time polymerase chain reaction

RNA from chondrocytes and human cartilage was isolated using RNeasy Mini kit (Qiagen, Valencia, CA, USA) according to the instructions. PCR was performed using SYBR Green QPCR Master Mix (Takara) with a Light Cycler apparatus (Bio-Rad, CFX-Touch). The PCR cycling consisted of 40 cycles of amplification of the template DNA with an annealing temperature of 60 °C. The relative level of expression of each target gene was calculated using the 2^−ΔΔ^Ct method. The amplification efficiencies of each primer pair were validated to enable quantitative comparison of gene expression. Each real-time PCR was performed on at least 3 different experimental samples. Representative results are displayed as target gene expression normalized to the reference gene actin. Error bars represent one SD from the mean of technical replicates. All primer sequences (Invitrogen Inc., Carlsbad, CA, USA) were designed using primer 5.0 software. The following primer sequences were utilized: 5′-CGAGTGGAAGAGCGGAGACT-3′ and 5′-AACTTTCATG GCGTCCAAGGT-3′ (Mouse *Col2a1*); 5′-GAAGAGCCTCGAATCACCTG-3′ and 5′-ATCCTG GGCACATTATGGAA-3′ (Mouse *Acan*); 5′-ATCCAGC TAAGACACAGCAAGCCA-3′ and 5′-TGGAGCACAAA GGAGTGGTCTCAA-3′ (Mouse *Mmp13*); 5′-GCTACTG CACAGGGAAGAGG-3′ and 5′-TGCAT nATTTGGAAC CCATT-3′ (Mouse *Adamts-5*);5′-CTGGAAAAGCTGGTG AAAGG -3′ and 5′-GGCCTGGATAACCTCTGTGA-3′ (Human *COL2A1*); 5′-TCCCCTGCTATTTCATCGAC-3′ and 5′-CCAGCAGCACTACCTCCTTC-3′ (Human *ACAN*); 5′-AACATCCAAAAACGCCAGAC -3′ and 5′-GGAAGTTCTGGCCAAAATGA-3′ (Human *MMP13*); 5′-TACTTGGCCTCTCCCATGAC-3′ and 5′-TTTGGAC CAGGGCTTAGATG -3′ (Human *ADAMTS-5*);.

### OA-inducing anterior cruciate ligament transection (ACLT) surgery and intra-articular delivery of cordycepin

All experiments involving mice were performed with the approval of the Zhejiang University Ethics Committee. Eight-week-old male C57BL/6 mice were administered ACLT (anterior cruciate ligament transection) surgery to both knees. Immediately after surgery, the animals were returned to their individual cages without joint immobilization. The mice in group I received intra-articular injections with PBS (10 μl) as a control. Ten days after ACLT, intra-articular injections of CM+HAMA (10 μl, group II), cordycepin alone (5 mg/kg, group III), or CM-cordycepin (cordycepin concentration: 5 mg/kg) combined with HAMA hydrogel (10 μl, group IV) were administered to the mice once per week. Each group consisted of eight mice. The mice were sacrificed 4 and 8 weeks after ACLT. The knee joint on the operated hind limb was dissected, embedded in paraffin, and investigated by safranin-O staining and immunostaining.

### OARSI scoring of murine cartilage

Semi-quantitative histopathological grading was performed using a modified version of the Chambers scoring system [32, 33], which has been established by the OARSI histopathology initiative as the standard method for grading mouse cartilage degeneration [34]. Based on this system, paraffin sections from each sample were scored after safranin-O staining. Histological grading was performed in 4 areas: medial femoral condyle, medial tibial plateau, lateral femoral condyle, and lateral tibial plateau. The grades of three blinded observers for each section were averaged, after which the data from each group of mice were collated.

### Histological analysis, immunohistochemistry and immunofluorescence of human cartilage, murine knee joints and mouse chondrocytes

The isolated knee joints were processed for histology and immunohistochemistry. Tissue samples were fixed in 4% (v/v) neutral buffered formalin for 24 h and decalcified in neutral 10% EDTA solution for 1 month at room temperature. Subsequently, the samples were dehydrated in an alcohol gradient, cleared, and embedded in paraffin blocks. Histological sections (8 μm) were prepared using a microtome. Six representative sections of each joint from various depths were mounted on slides, stained with safranin-O, and photographed digitally under a microscope. Human cartilage sections (8 μm) were prepared using a freezing microtome. After overnight incubation at 4°C with primary antibodies for MMP13 (Santa Cruz, sc30073), COL2A1(Abcam, ab116242), ADAMTS-5 (Santa Cruz, sc83186), and AGGRECAN (Santa Cruz, sc25674), the histological sections were incubated with secondary antibodies (Beyotime Institute of Biotechnology Inc., Jiangsu, China) for 2 h at room temperature. The DAB substrate system (Zsbio, Beijing, China) was used for color development. Hematoxylin staining was utilized to reveal the nuclei of the cells.

Mouse sections or chondrocytes evaluated by immunofluorescence were blocked by incubation with 5% (w/v) BSA, incubated with primary antibodies for LC3 (Sigma, L7543), incubated with corresponding secondary antibodies conjugated to Alexa Fluor 488 fluorescent dye (Invitrogen), and stained with DAPI (Beyotime Institute of Biotechnology). After staining, the histological sections were viewed under a microscope. The number of positively stained cells on the entire articular surface (including the femoral condyle and tibial plateau area) per specimen was counted in five sequential sections per joint in each group.

### Statistical analysis

All quantitative data are presented as mean ± SD. Histological grades are non-parametric ordinal ranks, and thus the OARSI scores among different groups were compared using Kruskal-Wallis analysis with the Mann-Whitney *U* test. Other statistical data were analyzed using one-way ANOVA/Tukey's post-hoc test. For all experiments, *P* < 0.05 was considered to be significant and is indicated by *P < 0.01 is indicated by **.

## CONCLUSIONS

In this study, we demonstrated that the combination of cordycepin encapsulated by CMs and photo-crosslinked HAMA hydrogel ameliorated the progression of surgically-induced osteoarthritis. These results demonstrate the importance of autophagy as a therapeutic target for OA prevention and treatment.

## SUPPLEMENTARY MATERIALS TABLES


