# Supplementary material for: Photo-crosslinked HAMA hydrogel with cordycepin encapsulated chitosan microspheres for osteoarthritis treatment
Source: Oncotarget. 2016 Dec 1;8(2):2835–49. doi: 10.18632/oncotarget.13748 (PMC5356846; doi:10.18632/oncotarget.13748)
Supplement: Supplementary file 1 [file oncotarget-08-2835-s001.pdf]

Photo-crosslinked HAMA hydrogel with cordycepin encapsulated chitosan microspheres for osteoarthritis treatment

Supplementary Materials

Supplementary Table S1: The data for the In vitro degradation of the hydrogel

	time (h)										
**Hydrogel mass (%)**	**0**	**1**	**2**	**3**	**4**	**5**	**6**	**7**	**8**	**9**	**10**
1	100	89.4	81.5	72.3	64.8	59.3	53.2	48	43	41	38
2	100	88.6	80.4	71.7	65.3	59.7	53.1	47.8	42.8	40.8	37.8
3	100	91.2	82.2	72.5	64.4	59.1	52.8	48.1	43	41.2	38.1

	time (h)										
**Hydrogel mass (%)**	**11**	**12**	**13**	**14**	**15**	**16**	**17**	**18**	**19**	**20**	**21**
1	36	33.1	32	31	30	29.3	29	27	27.5	26.3	25.1
2	35.9	33	32.1	30.9	30.1	29.3	28.9	27.2	27.3	26.2	24.8
3	36.2	33.1	32.2	31	30	29.2	29	26.8	27.5	26.3	25.3

	time (h)										
**Hydrogel mass (%)**	**22**	**23**	**24**	**25**	**26**	**27**	**28**	**29**	**30**	**31**	**32**
1	24.2	23.1	22.6	22.1	21.4	21.1	20.5	20.1	19.8	19.4	19.3
2	24.1	23.1	22.5	22	21.4	21	20.4	20	19.8	19.4	19.2
3	24.3	23	22.6	22.2	21.5	21.1	20.5	20.1	19.7	19.5	19.3

	time (h)										
**Hydrogel mass (%)**	**33**	**34**	**35**	**36**	**37**	**38**	**39**	**40**	**41**	**42**	**43**
1	18.9	18.3	17.7	17.3	17.1	16.3	16	15.8	15.3	14.9	14.4
2	18.9	18.3	17.6	17.3	17	16.2	16	15.8	15.3	14.8	14.3
3	18.8	18.2	17.7	17.2	17.1	16.3	15.9	15.7	15.2	14.9	14.4

	time (h)				
**Hydrogel mass (%)**	**44**	**45**	**46**	**47**	**48**
1	13.9	13.3	13	12.6	12.2
2	13.8	13.4	12.8	12.4	12
3	13.9	13.3	13	12.6	12.1
